# Mesenchymal Stem Cell-derived Extracellular Vesicles Prevent Experimental Bronchopulmonary Dysplasia Complicated By Pulmonary Hypertension

**DOI:** 10.1093/stcltm/szac041

**Published:** 2022-06-27

**Authors:** Mayank Sharma, Michael A Bellio, Merline Benny, Shathiyah Kulandavelu, Pingping Chen, Chawisa Janjindamai, Chenxu Han, Liming Chang, Shanique Sterling, Kevin Williams, Andreas Damianos, Sunil Batlahally, Kaitlyn Kelly, Daniela Aguilar-Caballero, Ronald Zambrano, Shaoyi Chen, Jian Huang, Shu Wu, Joshua M Hare, Augusto Schmidt, Aisha Khan, Karen Young

**Affiliations:** Department of Pediatrics, University of Miami Miller School of Medicine, Miami, FL, USA; Batchelor Children’s Research Institute, University of Miami Miller School of Medicine, Miami, FL, USA; Interdisciplinary Stem Cell Institute, University of Miami Miller School of Medicine, Miami, FL, USA; Department of Pediatrics, University of Miami Miller School of Medicine, Miami, FL, USA; Batchelor Children’s Research Institute, University of Miami Miller School of Medicine, Miami, FL, USA; Department of Pediatrics, University of Miami Miller School of Medicine, Miami, FL, USA; Interdisciplinary Stem Cell Institute, University of Miami Miller School of Medicine, Miami, FL, USA; Department of Pediatrics, University of Miami Miller School of Medicine, Miami, FL, USA; Batchelor Children’s Research Institute, University of Miami Miller School of Medicine, Miami, FL, USA; Department of Pediatrics, University of Miami Miller School of Medicine, Miami, FL, USA; Batchelor Children’s Research Institute, University of Miami Miller School of Medicine, Miami, FL, USA; Batchelor Children’s Research Institute, University of Miami Miller School of Medicine, Miami, FL, USA; Batchelor Children’s Research Institute, University of Miami Miller School of Medicine, Miami, FL, USA; Department of Pediatrics, University of Miami Miller School of Medicine, Miami, FL, USA; Batchelor Children’s Research Institute, University of Miami Miller School of Medicine, Miami, FL, USA; Department of Pediatrics, University of Miami Miller School of Medicine, Miami, FL, USA; Batchelor Children’s Research Institute, University of Miami Miller School of Medicine, Miami, FL, USA; Department of Pediatrics, University of Miami Miller School of Medicine, Miami, FL, USA; Batchelor Children’s Research Institute, University of Miami Miller School of Medicine, Miami, FL, USA; Department of Pediatrics, University of Miami Miller School of Medicine, Miami, FL, USA; Batchelor Children’s Research Institute, University of Miami Miller School of Medicine, Miami, FL, USA; Department of Pediatrics, University of Miami Miller School of Medicine, Miami, FL, USA; Batchelor Children’s Research Institute, University of Miami Miller School of Medicine, Miami, FL, USA; Department of Pediatrics, University of Miami Miller School of Medicine, Miami, FL, USA; Batchelor Children’s Research Institute, University of Miami Miller School of Medicine, Miami, FL, USA; Department of Pediatrics, University of Miami Miller School of Medicine, Miami, FL, USA; Batchelor Children’s Research Institute, University of Miami Miller School of Medicine, Miami, FL, USA; Department of Pediatrics, University of Miami Miller School of Medicine, Miami, FL, USA; Batchelor Children’s Research Institute, University of Miami Miller School of Medicine, Miami, FL, USA; Department of Pediatrics, University of Miami Miller School of Medicine, Miami, FL, USA; Batchelor Children’s Research Institute, University of Miami Miller School of Medicine, Miami, FL, USA; Department of Pediatrics, University of Miami Miller School of Medicine, Miami, FL, USA; Batchelor Children’s Research Institute, University of Miami Miller School of Medicine, Miami, FL, USA; Interdisciplinary Stem Cell Institute, University of Miami Miller School of Medicine, Miami, FL, USA; Department of Medicine, University of Miami Miller School of Medicine, Miami, FL, USA; Department of Pediatrics, University of Miami Miller School of Medicine, Miami, FL, USA; Batchelor Children’s Research Institute, University of Miami Miller School of Medicine, Miami, FL, USA; Interdisciplinary Stem Cell Institute, University of Miami Miller School of Medicine, Miami, FL, USA; Department of Pediatrics, University of Miami Miller School of Medicine, Miami, FL, USA; Batchelor Children’s Research Institute, University of Miami Miller School of Medicine, Miami, FL, USA; Interdisciplinary Stem Cell Institute, University of Miami Miller School of Medicine, Miami, FL, USA

**Keywords:** mesenchymal stem cell, extracellular vesicles, preterm, pulmonary hypertension, bronchopulmonary dysplasia

## Abstract

Mesenchymal stem cell (MSC) extracellular vesicles (EVs) have beneficial effects in preclinical bronchopulmonary dysplasia and pulmonary hypertension (BPD-PH) models. The optimal source, dosing, route, and duration of effects are however unknown. The objectives of this study were to (a) compare the efficacy of GMP-grade EVs obtained from Wharton’s Jelly MSCs (WJ-MSCs) and bone marrow (BM-MSCs), (b) determine the optimal dosing and route of administration, (c) evaluate its long-term effects, and (d) determine how MSC EVs alter the lung transcriptome. Newborn rats exposed to normoxia or hyperoxia (85% O_2_) from postnatal day (P)1-P14 were given (a) intra-tracheal (IT) BM or WJ-MSC EVs or placebo, (b) varying doses of IT WJ-MSC EVs, or (c) IT or intravenous (IV) WJ-MSC EVs on P3. Rats were evaluated at P14 or 3 months. Early administration of IT BM-MSC or WJ-MSC EVs had similar beneficial effects on lung structure and PH in hyperoxia-exposed rats. WJ-MSC EVs however had superior effects on cardiac remodeling. Low, medium, and high dose WJ-MSC EVs had similar cardiopulmonary regenerative effects. IT and IV WJ-MSC EVs similarly improved vascular density and reduced PH in hyperoxic rats. Gene-set enrichment analysis of transcripts differentially expressed in WJ-MSC EV-treated rats showed that induced transcripts were associated with angiogenesis. Long-term studies demonstrated that a single early MSC EV dose has pulmonary vascular protective effects 3 months after administration. Together, our findings have significant translational implications as it provides critical insight into the optimal source, dosing, route, mechanisms of action, and duration of effects of MSC-EVs for BPD-PH.

## Graphical Abstract

**Graphical Abstract:**
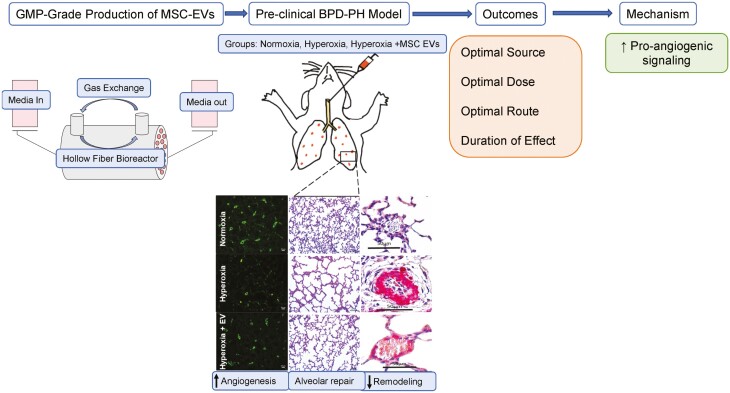

**Significance Statement**

Mesenchymal stem cell (MSC)-derived extracellular vesicles (EVs) are a potential strategy to reduce bronchopulmonary dysplasia (BPD) complicated by pulmonary hypertension (PH). Our study provides comprehensive insight into the ideal source, dosing, route, and long-term effects of GMP-grade MSC EVs for BPD and PH. We show that bone marrow (BM)-MSC and Wharton’s Jelly (WJ)-MSC EVs have similar lung protective effects in experimental BPD and PH, and that systemically and locally delivered WJ-MSC EVs attenuate PH to a similar degree. Importantly, we also show that the cardiopulmonary protective effects of a single neonatal dose of WJ-MSC EVs persist into young adulthood.

## Background

Bronchopulmonary dysplasia (BPD) is one of the most common complications in extreme preterm infants and pulmonary hypertension (PH) is a major co-morbidity contributing to worse outcomes.^1,2^ Emerging evidence shows that 15–25% of infants with BPD develop early PH and this rate is higher with increasing BPD severity.^3-7^ Infants with BPD and PH have disrupted pulmonary vascular development and early signs of pulmonary vascular disease at 7 days of age are associated with an increased risk of BPD and PH at 36 weeks postmenstrual age.^7^ Multiple prenatal and postnatal factors contribute to the disordered angiogenesis, alveolar simplification, and pulmonary vascular remodeling, seen in BPD and PH.^8^ However, because of this multifactorial etiology, there are limited therapeutic interventions to prevent PH in BPD patients.^9^

Mesenchymal stem cells (MSCs) are multipotent progenitor cells with anti-inflammatory, pro-angiogenic, anti-apoptotic, and anti-fibrotic properties.^10^ In pre-clinical BPD models, MSCs reduce inflammation, improve lung angiogenesis, and restore lung structure.^11-13^ Importantly, MSCs also attenuate PH and reduce pulmonary vascular remodeling in severe BPD models.^12,14-16^ Early clinical trials show that MSCs administered to preterm infants at high risk for BPD are safe and effective.^17^ Yet, as MSC therapy for the prevention of BPD, moves to the bedside, our group and others have demonstrated that MSC-conditioned media have comparable or superior efficacy to MSCs themselves in preclinical BPD models.^15,18,19^ Robust evidence in other disease models also shows that MSCs exert their regenerative effects in a cell-autonomous manner through paracrine-mediated mechanisms.^20,21^

Extracellular vesicles (EVs) are a heterogenous group of nanoparticles released by all cells including MSCs. They are broadly categorized based on their diameter, ranging in size between 30 and 1000 nm.^22^ EVs contain a cargo of proteins, lipids, mRNA, and microRNA that play a critical role in intercellular communication.^23^ Studies using both human bone marrow (BM-MSCs) and Wharton’s Jelly MSCs (WJ-MSCs) derived EVs in experimental BPD models have shown them to be effective in restoring alveolar structure and alleviating PH.^18,24,25^ Yet, one of the challenges for transitioning MSC EVs from the bench to the bedside is the generation of high quantity good manufacturing practices (GMP)-compliant products as well as optimization of dosing, route, and source of MSC EVs.^20^ To date, there has been one published study testing a single dose of clinical-grade WJ-MSC EVs in an animal model of BPD but the effect on PH was not assessed.^26^ There is also limited data on the ideal source and long-term effects of MSC EVs in BPD models.

The objectives of our study were to compare the efficacy of GMP-grade IT BM-MSC and WJ-MSC EVs in preserving lung structure and preventing PH in a severe BPD model, elucidate the optimal dosing and route of MSC EVs to prevent BPD and PH and determine whether a single dose of MSC EVs administered in the neonatal period has long term cardiopulmonary protective effects.

## Materials and Methods

### WJ-MSC Production

WJ-MSCs were expanded from umbilical cords obtained with written informed consent and approval by the Institutional Review Board of the University of Miami (IRB number 20100986). Wharton’s Jelly (WJ) isolated from a human umbilical cord obtained from a healthy and full-term infant was first cut into small pieces. The pieces were placed into several Petri dishes with a minimum medium to allow attachment. The dishes were placed into a 37ºC incubator with 5% CO_2_. The growth medium was composed of 20% fetal bovine serum in α-MEM. When passage 1 cells reached confluence, the cells were harvested and cryopreserved at 2 to 5 × 10^6^ cells/mL of cryopreservation medium. The cryopreservation medium used was CryoStor CS10 (BioLife Solutions, Bothell, WA). Flow cytometry analysis with CD31, CD45, CD105, CD90 antibodies, and isotype control was performed on P2 WJ-MSC to confirm the MSC identity.

### BM-MSC Production

BM-MSCs were isolated from fresh adult bone marrow aspirates, and obtained from a commercial source (AllCells, Alameda, CA). Briefly, bone marrow was processed using a lymphocyte separation medium (LSM; MediaTech Inc, Manassas, VA) to prepare density-enriched, mononuclear cells (MNCs). The cells were diluted with Plasma-Lyte A (Baxter, Deerfield, IL) and layered onto LSM using conical tubes to isolate MNCs following established standard operating procedures. The MNCs were washed with Plasma-Lyte A containing 1% human serum albumin (Baxter, Deerfield, IL). The washed cells were sampled to determine the total number of viable nucleated cells. The MNCs were cultured at P0 and when confluent, passaged to P1 in a 37ºC incubator with 5% CO_2_. The growth medium was composed of 5% PLTMax (Millcreek, Rochester, MN), heparin, and 1% penicillin/streptomycin in α-MEM.

### MSC EV Production

WJ-MSCs and BM-MSCs were collected at P1 harvests and expanded in a Quantum Bioreactor (Terumo BCT, Lakewood, CO) for P2 expansion and EV harvest. To begin the bioreactor expansion, the Quantum bioreactors were primed with PBS and coated with 5 mg of human fibronectin (Corning, Tewksbury, MA) diluted in 100 mL of PBS. Fibronectin coating was performed for a minimum of 4 h followed by a system washout with expansion media supplement with 20% FBS (WJ-MSC expansion) or 5% PLTMax (BM-MSC expansion). After expansion media exchange, 30.0 × 10^6^ MSCs were loaded and allowed to attach for 24 h. Once cell attachment was completed, cell expansion was performed by increasing the daily media input feeding rate to compensate for the growing number of cells. A daily sample of the outer loop media was collected to test daily lactate production and to estimate the total number of growing cells. After peak expansion was obtained, expansion media was washed away with inner and outer loop washouts with PBS. After completion of the washout cycle, wash media was replaced with α-MEM without supplementation. Media conditioning was then allowed to continue for 120 h and inner loop outlet waste media was collected daily for EV isolation. Every 24 h, 200 mL of conditioned media was collected from the Quantum, and EVs were collected. Harvested conditioned media was subjected to sequential centrifugation at 2000 x *g* for 30 min and 20 000 × *g* for 30 min followed by EV precipitation by ultracentrifugation (Beckman Coulter) at 100 000 × *g* for 70 min using a Ti70 rotor. EV pellets were resuspended in 4–5 mL of saline.

### Nanosight Analysis

Nanosight nanoparticle analysis was performed on the final EV products using the Nanosight NS300 instrument (Malvern Panalaytical) and NTA 3.3 Dev Build 3.3.104 software. Mean concentrations and mode size were determined from 5 videos taken of 1 sample analyzed at a 1:1000 dilution, pump speed 30, and video length 30 s.

### Animal Maintenance

Pregnant Sprague Dawley rats were purchased from Charles River Laboratories (Wilmington, MA). Rats were treated according to the National Institutes of Health guidelines for the use and care of laboratory animals following approval of the study protocol by the University of Miami Animal Care and Use Committee.

### Experimental BPD and PH Protocol

Newborn Sprague Dawley rats were assigned to room air (21% O_2_) or hyperoxia (85% O_2_) from postnatal day (P) 1 to 14. Oxygen exposure was achieved in a Plexiglass chamber by a flow-through system and the oxygen level inside the chamber was monitored daily with a Maxtec oxygen analyzer (Model OM25-RME; Maxtec, Salt Lake City, Utah). Dams were rotated every 48 h between hyperoxia and normoxia chambers to prevent damage to their lung. Litter size was adjusted to 10-12 pups to control for the effect of litter size on nutrition and growth. Hemodynamic, lung morphometric, and molecular studies were performed at P14. To study the long-term effects of MSC EVs, rats exposed to room air or hyperoxia from P1–14 were maintained in room air until 3 months of life and evaluated at this time.

### MSC EV Administration

To compare the efficacy of BM-MSC and WJ-MSC EVs, newborn rats exposed to room air or hyperoxia from P1-P14 were given a single intra-tracheal (IT) injection of 12 × 10^8^ particles per gram body weight of BM-MSC and WJ-MSC EVs suspended in 50 µL saline, or saline as placebo (PL) on P3 as previously described.^15^ Briefly, following sedation with isoflurane, the trachea was exposed through a small midline incision in the neck. MSC EVs or PL were delivered by direct tracheal puncture with a 30-gauge needle. Rats were placed in a warmed plastic chamber under normoxic or hyperoxic conditions for recovery. Once the rats were fully awake, they were returned to their dams.

To determine the optimal dose of MSC EVs, 3 different doses of IT delivered WJ-MSC EVs or PL were injected on P3 to newborn rats exposed to normoxia or hyperoxia from P1–P14: 2 × 10^8^ particles per gram body weight (low dose); 12 × 10^8^ particles per gram body weight (medium dose); 60 × 10^8^ particles per gram body weight (high dose).

To determine the ideal route of administration, IT or intravenous (IV) WJ-MSC EVs (medium dose) or PL was administered on P3. Briefly, following sedation with isoflurane, IV WJ-MSC EVs or PL was given by direct injection into the superficial temporal vein as previously described.^27^

To determine the long-term effects of IT WJ-MSC EVs, newborn rats exposed to room air or hyperoxia from P1–P14 were given a single IT medium WJ-MSC EV dose on P3. Rats were studied at 3 months of life.

### Verification of IT MSC EV Lung Delivery

 MSC EVs or PL labeled with a fluorescent dye (ExoGlow -Vivo EV Labeling Kit, System Biosciences, Palo Alto, CA) were injected with IT on P3. Saline incubated with free dye and processed identically as labeled EVs was used as PL. The animals were imaged at various time points following IT injection of labeled EVs and PL using an in vivo imaging system (IVIS in-vivo Imaging System; PerkinElmer, Waltham, MA). Rats injected with labeled EVs or PL were also euthanized 90 min after injection and organs were removed and imaged under IVIS.

### Hemodynamic Studies

Following room air and hyperoxic exposures, rats were evaluated at P14 or 3 months of life. Rats were anesthetized with 1% isoflurane and right ventricular systolic pressure (RVSP) was measured as previously described.^28^ Briefly, after thoracotomy, a 25-gauge needle fitted to a pressure transducer was inserted into the right ventricle. RVSP was measured and continuously recorded on a Gould polygraph (model TA-400, Gould instruments, Cleveland, Ohio). Immediately after RVSP measurements were obtained, the animals were euthanized. Right ventricular hypertrophy (RVH) was determined by measuring the weight ratio of the right ventricle (RV) to the left ventricle (LV) and septum (S).

### Lung Morphometric Analysis

Lungs were perfused and fixed in 4% paraformaldehyde and embedded in paraffin. Serial sections (5 μm) thick were taken from the upper and lower lobes and stained with hematoxylin and eosin. Images from 5 randomly selected, non-overlapping parenchymal fields were acquired from lung sections of each animal using an Olympus Qcolor 3 color camera interfaced with a light microscope (Model Leica DMI 4000B) at 10× magnification. Alveolarization was determined by calculating mean linear intercept (MLI) and radial alveolar count (RAC). MLI was calculated by determining the average distance between intersects of alveolar septal tissue and a superimposed counting grid. RAC was determined by counting the number of alveoli between the pleural surface of the lung and the nearest terminal bronchiole.

### Pulmonary Vascular Density

Lung sections were de-paraffinized, rehydrated, and stained with polyclonal rabbit anti-human Von Willebrand Factor (vWF, 1:50; Dako Corp, Carpinteria, CA). The number of vessels (20-50 μm diameter) per high power field (HPF) was quantified in 5 randomly selected, non-overlapping, parenchymal fields from the lung sections of each animal.

### Pulmonary Vascular Remodeling

Paraffin-embedded sections were stained with polyclonal rabbit anti-human vWF and monoclonal mouse anti-α-smooth muscle actin (α-SMA: 1:500, Sigma–Aldrich; St. Louis, MO). Medial wall thickness (MWT) of partially and fully muscular arteries (20-50 μm) was determined by using the formula: 2(MT) × 100/ED, where MT is the distance between the internal and external boundaries of the α-SMA layer and ED is the external diameter. All analyses were performed by a blinded observer.

### Matrigel Assay

Human pulmonary microvascular endothelial cells (HULECs: ATCC, Manassas, VA), passage 3–4, were seeded at 2 × 10^4^ cells/well into 96 well plates. HULECs incubated with varying doses of WJ-MSC EVs (10 and 20 ng/mL), or EV-depleted media were cultured in normoxic (21% O_2_, 5% CO_2_) or hyperoxic (95% O_2_, 5% CO_2_) conditions for 24 h. The effect of WJ-MSC EVs on capillary tube formation was determined by matrigel assay as previously described.**^29^** Bright field images were taken at 5, 10, and 24 h. All experiments were done in quadruplicate and tube formation was assessed using the ImageJ Angiogenesis Analyzer.

### RNA Isolation and Sequencing

Total RNA was extracted from frozen lung tissues of P14 rats with a miRNeasy kit (QIAGEN, Valencia, CA) per the manufacturer’s protocol. RNA quality and integrity were verified with the Agilent Tapestation Bioanalyzer (Agilent, Santa Clara, CA) and all samples had a RNA integrity number greater than 8. For lung tissues, RNA sequencing was performed by the Sequencing Core at the Center for Genome Technology, John P Hussman Institute for Human Genomics, University of Miami Miller School of Medicine. Sequencing was performed with a real depth of 33–48 million reads per sample for 100 bp single-end reads on the Illumina NovaSeq 6000 platform (Illumina, San Diego, CA). The adapter content was trimmed with Trimomatic.^30^ Raw sequence reads in FASTQ format were pseudoaligned to the rat genome (Rattus norvegicus, build rn6.0) and quantified using Kallisto.^31^ Differential expression analyses were performed using DESeq2.^32^ Genes were considered differentially expressed based on their *P*-value (< .05) and *q*-value (<0.1). Lists of differentially expressed transcripts were used for functional enrichment analysis of Gene ontology and pathway terms on the ToppCluster web server.^33^ For each group-pair comparison, we reported only unique terms associated with either induced or suppressed transcripts and with at least 2 associated transcripts. Negative log *P*-values represent terms associated with suppressed transcripts and positive log *P*-values are associated with induced transcripts. We performed pathway and upstream analysis on differentially expressed transcripts with IPA (QIAGEN Inc., https://www.qiagenbioinformatics.com/products/ingenuitypathway-analysis). Average transcript expression was used for hierarchical clustering of differentially expressed transcripts from all contrasts. Based on expression pattern, we identified 5 unique clusters and performed functional enrichment analysis for clusters of interest using ToppCluster^33^ to identify uniquely regulated biological processes, molecular functions, cellular components, and pathways associated with the clusters. We also performed pathway and upstream analysis on clusters of interest using the IPA platform.

To determine whether MSC EVs alter the gene expression of pro-angiogenic genes in hyperoxia-exposed HULECs, we also evaluated the mRNA expression of the following pro-angiogenic genes: endothelial nitric oxide synthase (eNOS), vascular endothelial growth factor (VEGF), hepatocyte growth factor (HGF), angiopoietin-1 (Ang-1), and chemokine receptor 4 (CXCR4) using an ABI Fast 7500 System (Applied Bio-Rad, Foster City, CA). HULECs incubated with WJ-MSC EVs (20 ng/ml), or EV-depleted media were cultured in normoxic (21% O_2_, 5% CO_2_) or hyperoxic (95% O_2_, 5% CO_2_) conditions for 6 h. Primers for human eNOS, VEGF, HGF, Ang-1, CXCR4, and GAPDH were pre-developed by Applied Biosystems. For each target gene, a standard curve was established by performing a series of dilutions of the first-strand cDNA. The mRNA expression of target genes was normalized to GAPDH. Experiments were performed in triplicate.

### Lung Cytokine Analyses

Multiplex bead-based assay (Eve Technologies (Calgary, AB, Canada) was used to quantify lung cytokine and chemokine levels.

### Statistical analyses

Data reported as mean ± SEM were analyzed by 2-way ANOVA with post hoc Holm-Sidak test using Sigma Stat software. For survival analysis, data shown in Kaplan-Meier curves were compared with the log-rank test. *P* < .05 were considered statistically significant.

## Results

### Effective Lung Delivery of IT Administered MSC EVs

**Figure 1.**
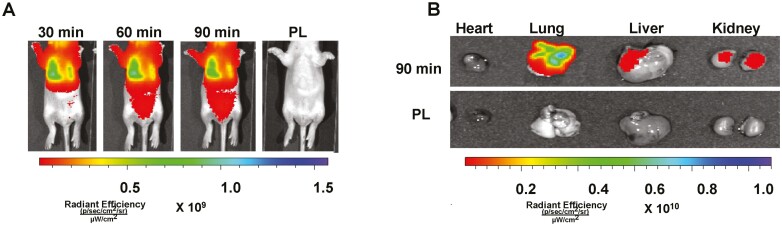
Effective delivery of intra-tracheal (IT) MSC extracellular vesicles (EVs). (**A**) Representative bioluminescence images of neonatal rats taken 30, 60, and 90 minutes following IT administration of fluorescent labeled MSC EVs or placebo (PL). (**B**) Ex vivo imaging performed 90 minutes post-IT injection shows increased fluorescent signal in the lungs, kidney and liver.

Neonatal rats that received IT fluorescent labeled MSC EVs had significant bioluminescence in the thoracic region (Fig. 1A), and this was persistent, 90 min post-injection. Moreover, as compared to animals who received IT PL, ex vivo imaging showed significant bioluminescence in the lungs, kidneys, and liver of neonatal rats, (Fig. 1B).

### Influence of MSC Source on EV Efficacy in BPD and PH

**Figure 2.**
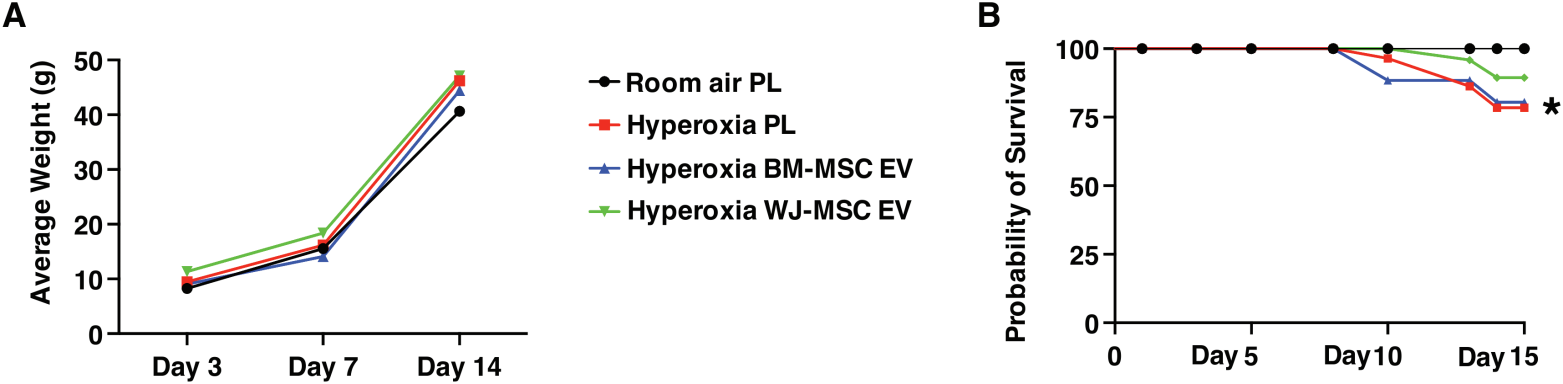
Effect of BM and WJ-MSC EVs on growth and survival. (**A**) No difference in growth velocity among room air and hyperoxia groups. (**B**) Kaplan-Meier survival curve showing differences in survival between room air and hyperoxia exposed rats. Compared to room air placebo (PL) rats, there was significantly increased mortality of hyperoxia PL-treated rats, *P < .05; room air PL vs. hyperoxia PL; *N* = 9/group. No difference in survival among the hyperoxia groups.

**Figure 3.**
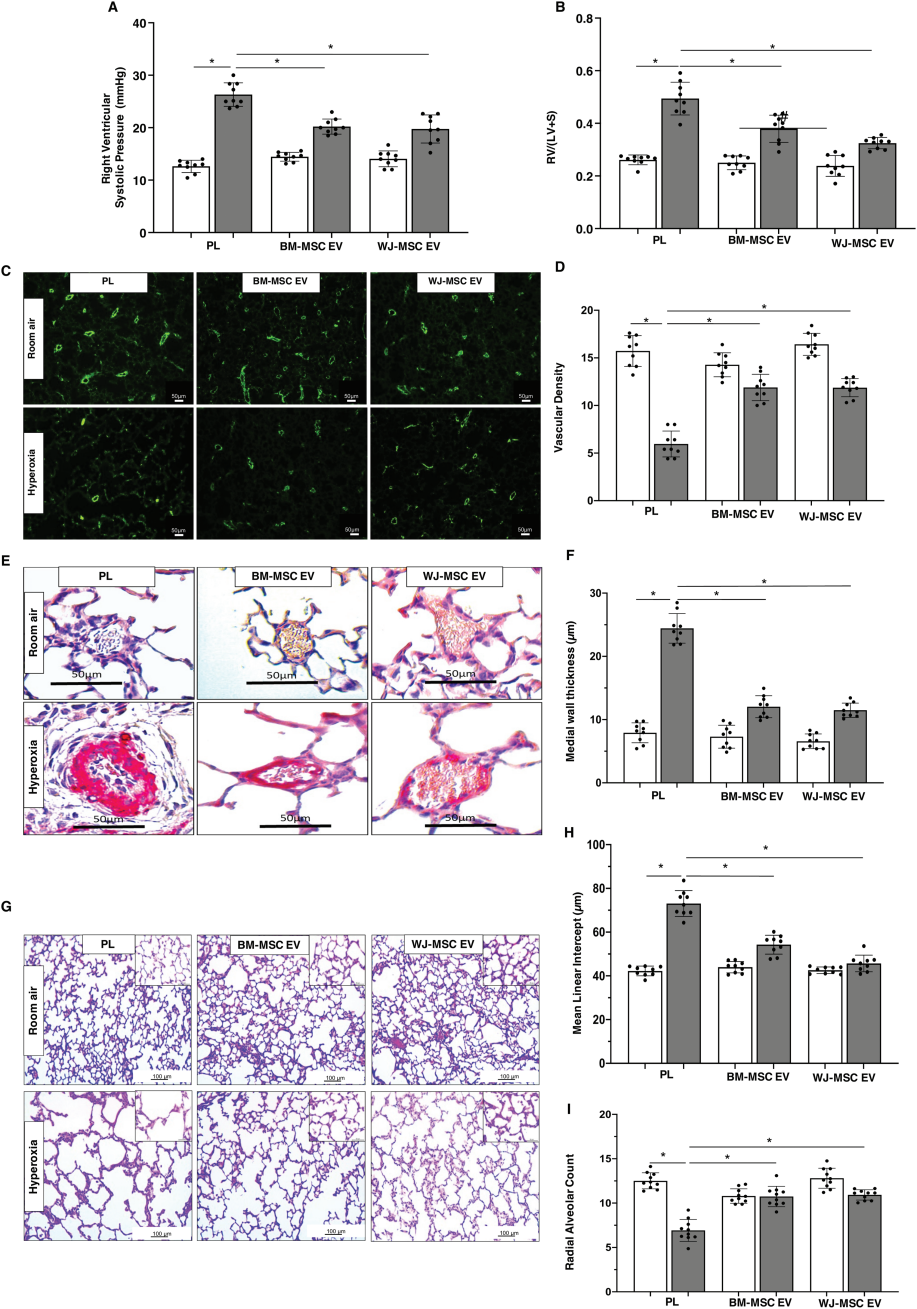
BM and WJ-MSC EVs have similar effects in BPD and PH. Both IT BM-MSC and WJ-MSC EVs significantly reduced (**A**) right ventricular systolic pressure and (**B**) weight ratio of the right ventricle to left ventricle + septum (RV/LV+S) but the reduction in RV/LV+S was greater in 2 week old hyperoxia exposed rats who received WJ-MSC EVs. (**C**) Representative lung sections stained with Von Willebrand Factor showing increased vessels in both IT hyperoxia BM-MSC and WJ-MSC EV treated groups. Original magnification 10×. (**D**) Similar improvement in lung vascular density in both IT hyperoxia BM-MSC and WJ-MSC EV groups. (**E**) Lung sections stained with α-smooth muscle actin demonstrating decreased pulmonary vascular remodeling in hyperoxia-exposed rats treated with IT BM-MSC or WJ-MSC EV. Original magnification 40×. (**F**) Reduced medial wall thickness in hyperoxia-exposed rats treated with IT BM-MSC or WJ-MSC EVs. (**G**) H&E-stained lung sections demonstrating improved alveolar structure in hyperoxia-exposed rats that received IT BM-MSC or WJ-MSC EV. Original magnification 10×. Inset is 40×. Morphometric analysis showing (**H**) decreased mean linear intercept and (**I**) increased radial alveolar count in IT BM-MSC or WJ-MSC EV treated hyperoxia rats. Data are presented as mean ± SEM; *N* = 9/group. *P < .05; room air placebo (PL) vs. hyperoxia PL or hyperoxia PL vs. hyperoxia BM-MSC or WJ-MSC EV. ^#^*P* < .05, hyperoxia BM-MSC EV vs. hyperoxia WJ-MSC EV. Room air: open bar; hyperoxia: gray bar.

There was no difference in the growth velocity among the groups (Fig. 2A). As compared to the RA PL rats, Kaplan Meier log-rank survival analysis demonstrated increased mortality of the hyperoxia PL rats (Fig. 2B). There was however no significant difference in survival rates among the hyperoxia PL, hyperoxia BM-MSC EV, or hyperoxia WJ-MSC EV groups, although there was a trend for less mortality in the hyperoxia WJ-MSC EV group (Fig. 2B). Hyperoxia exposed PL-treated rats developed PH as evidenced by significantly elevated RVSP and RV/LV+S (Fig. 3A, 3B). In contrast, both IT administration of BM-MSC and WJ-MSC EVs significantly reduced RVSP and there was no difference between the hyperoxia EV treated groups (Fig. 3A, 3B). In contrast, although both BM-MSC and WJ-MSC EVs reduced the degree of RVH, this was most pronounced in the hyperoxia WJ-MSC EV treated rats, (Fig. 3B).

Given that decreased angiogenesis and pulmonary vascular remodeling are key structural changes in the lungs of infants with severe BPD complicated by PH, we questioned whether the beneficial effects of BM-MSC and WJ-MSC EVs were secondary to an improvement in angiogenesis and or reduction in pulmonary vascular remodeling. Hyperoxia exposure significantly decreased lung vascular density, (Fig. 3C, 3D). Treatment with IT BM-MSC and WJ-MSC EVs restored lung vascular density and there was no significant difference between the hyperoxia EV treated groups, (Fig. 3C, 3D). Moreover, whereas hyperoxia-exposed PL-treated animals had increased medial wall thickness of pulmonary vessels, this was significantly reduced after IT administration of both MSC EVs with comparable efficacy between the BM-MSC and WJ-MSC EV groups, (Fig. 3E, 3F).

As most preterm infants with severe BPD and PH have arrested alveolar growth, we also evaluated the effects of the MSC EVs on lung alveolarization. Hyperoxia exposed PL-treated animals exhibited simplified alveolar structure and morphometric analysis demonstrated an increase in MLI, and a decrease in RAC, (Fig. 3H, 3I). IT administration of BM-MSC and WJ-MSC EVs prevented the arrest of alveolarization and there was no difference between the hyperoxia EV treated groups, (Fig. 3H, 3I).

### Optimal Effective Dose of MSC EVs in BPD and PH

**Figure 4.**
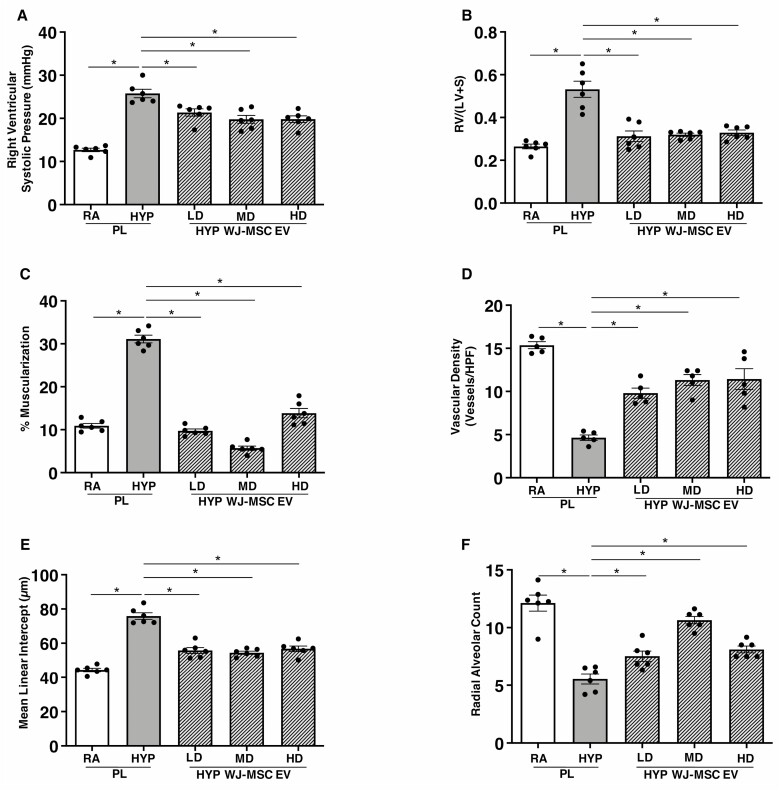
Non-dose-dependent effects of WJ-MSC EVs in BPD and PH. Similar reduction in (**A**) right ventricular systolic pressure, (**B**) RV/LV+S, and (**C**) percentage of muscularized vessels in 2-week-old hyperoxia (HYP) exposed rats treated with IT low dose (LD), medium dose (MD) and high dose (HD) WJ-MSC EV. IT WJ-MSC EV non-dose dependently (**D**) increased lung vascular density, (**E**) decreased mean linear intercept and (**F**) increased radial alveolar count. Data are presented as mean ± SEM; *N* = 6/group. *P < .05, room air (RA) placebo (PL) vs. HYP PL or HYP PL vs. HYP WJ-MSC EV LD, MD or HD.

Given the trend for better survival and logistical accessibility advantage, we performed a dose–finding study using only WJ-MSC EVs. We administered a single IT injection of either PL, low dose (2 × 10^8^ particles per gram body weight), medium dose (12 × 10^8^ particles per gram body weight), or high dose (60 × 10^8^ particles per gram body weight) WJ-MSC EVs on P3 to newborn rats exposed to RA or hyperoxia from P1-P14. Hyperoxia-PL treated rats had increased RVSP and RV/LV+S, (Figs. 4A, 4B), and treatment with WJ-MSC EVs non-dose dependently attenuated PH and RVH, (Figs. 4A, 4B). Lung vascular density, pulmonary vascular remodeling, and lung alveolar structure also significantly improved following administration of all 3 WJ-MSC EV doses and there was no dose–effect relationship (Figs. 4C–4F).

**Figure 5.**
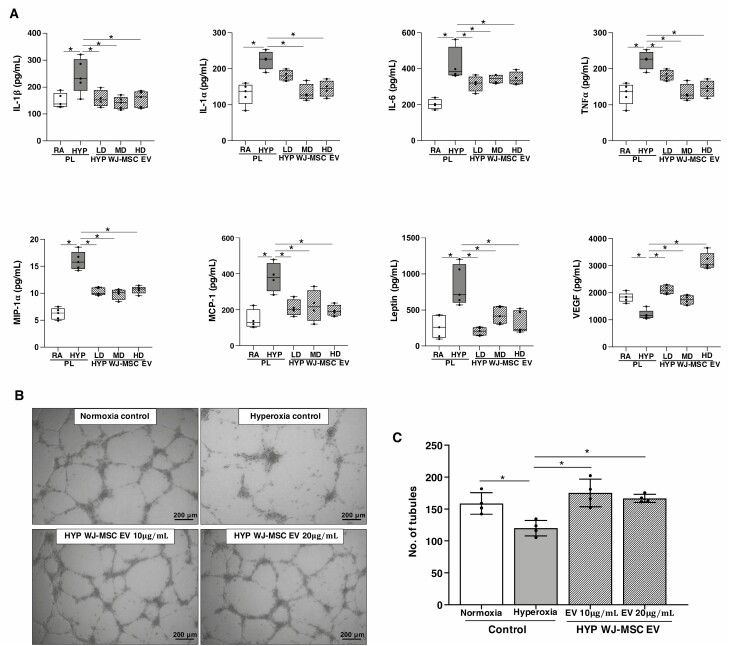
Non-dose-dependent effects of WJ-MSC EVs on lung cytokine concentration. Multiplex array demonstrate similar reduction in the lung concentration of (**A**) IL-1β, IL-1α, IL-6, TNF-α, MIP-1 α, MCP-1, and leptin concentration in 2-week-old hyperoxia (HYP) exposed rats treated with IT low dose (LD), medium dose (MD), and high dose (HD) WJ-MSC EV. In contrast, lung VEGF concentration was increased by all doses of WJ-MSC EV. Data are presented as mean ± SEM; *N* = 4–5/group. *P < .05, room air (RA) placebo (PL) vs. HYP PL or HYP PL vs. HYP WJ-MSC EV LD, MD, or HD. (**B**) Treatment of hyperoxia-exposed human pulmonary microvascular endothelial cells with WJ-MSC EVs 10 or 20 ug/mL increased capillary tube formation and (**C**) this was non-dose dependent. All experiments were performed in quadruplicate. *P < .05, normoxia control vs. hyperoxia control or hyperoxia control vs. hyperoxia WJ-MSC EV 10 or 20 ng/mL.

Since inflammation plays a crucial role in the pathogenesis of BPD and PH, we also evaluated whether the dose of WJ-MSC EVs would influence lung inflammation markers. Whereas hyperoxia-PL rats had increased concentration of interleukin-1β, interleukin-1α, interleukin-6, tumor necrosis factor-1α, macrophage inflammatory protein-1α, monocyte chemoattractant protein-1, and leptin in lung homogenates, hyperoxia-exposed rats that received WJ-MSC EVs had a non-dose-dependent reduction in the concentration of these cytokines (Fig. 5A).

We also evaluated the effect of WJ-MSC EVs on lung VEGF concentration in our experimental BPD and PH model. Interestingly, while low and medium-dose WJ-MSC EVs restored lung VEGF concentration to near normoxia levels, lung VEGF concentration in rats treated with high-dose WJ-MSC EVs trended higher than the RA-PL group (Fig. 5A). WJ-MSC EVs however had non-dose-dependent pro-angiogenic effects on hyperoxia-exposed HULECs (Fig. 5B–5C).

### Effects of Local and Systemically Delivered MSC EVs in BPD and PH

**Figure 6.**
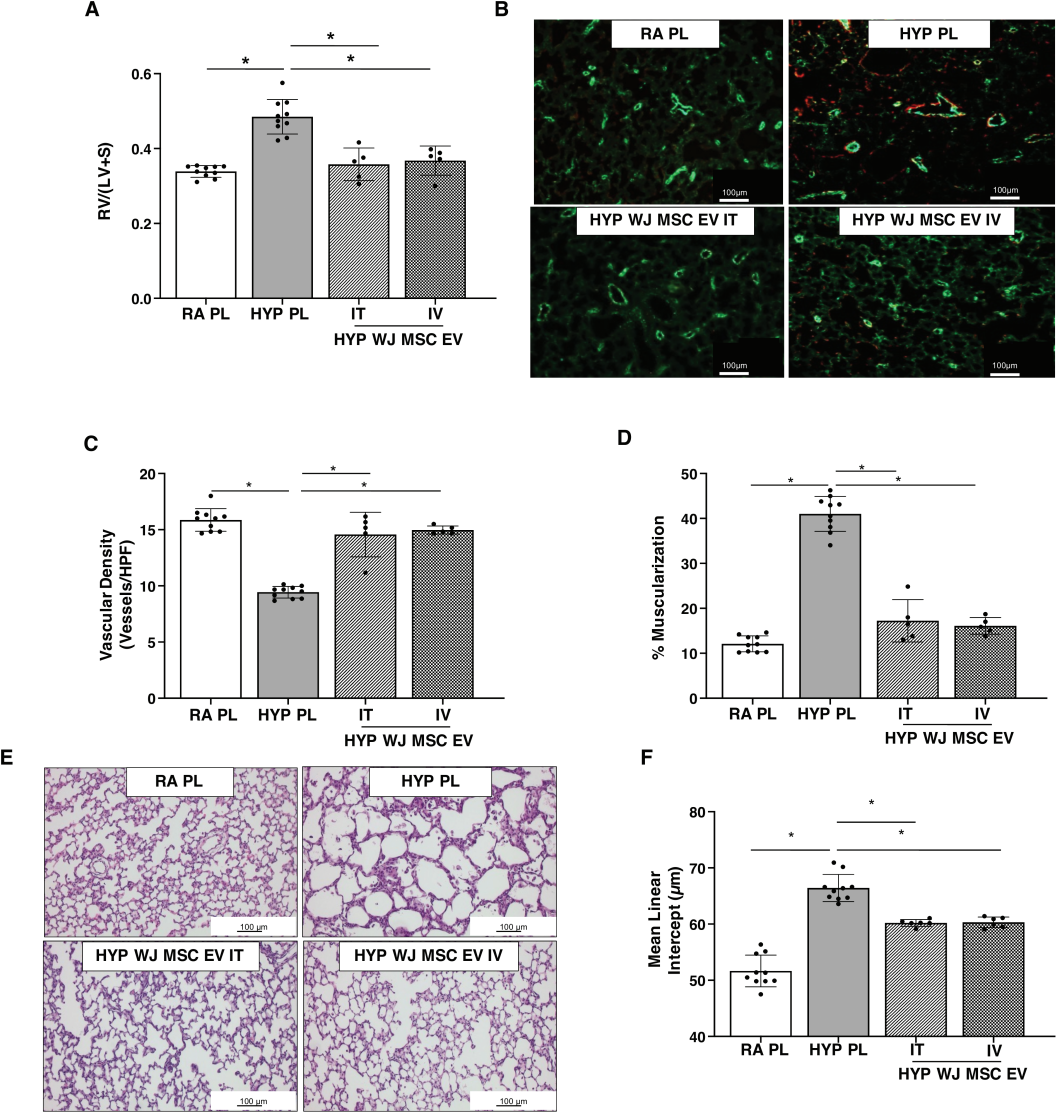
IV and IT delivered WJ-MSC EVs have similar lung protective effects in BPD and PH. IT and IV WJ-MSC EVs significantly reduced (**A**) weight ratio of the right ventricle to left ventricle + septum (RV/LV+S). (**B**) Representative lung sections stained with Von Willebrand Factor (green) and α-smooth muscle actin (red) showing increased vessels and decreased muscularization in both IV and IT hyperoxia WJ-MSC EV treated groups. Original magnification 10×. (**C**) Similar improvement in lung vascular density and (**D**) percentage of muscularized vessels in both IV and IT hyperoxia WJ-MSC EV groups. (**E**) H&E-stained lung sections demonstrating improved alveolar structure in hyperoxia-exposed rats that received IV or IT WJ-MSC EV. Original magnification 10×. Morphometric analysis showing (**F**) similarly decreased mean linear intercept in IT and IV WJ-MSC EV treated hyperoxia rats. Data are presented as mean ± SEM; *N* = 8–16/group. *P < .05; room air placebo (PL) vs. hyperoxia PL or hyperoxia PL vs. hyperoxia IT or hyperoxia IV WJ-MSC EV. Room air: open bar; hyperoxia: gray bar.

Prior studies have shown that IV and IT delivered MSC EVs have significant lung protective effects in experimental BPD, but to date, no comparative studies have been performed.^27,34^ We therefore administered a single IT or IV injection of either PL or medium-dose WJ-MSC EVs on P3 to newborn rats exposed to RA or hyperoxia from P1-P14. Rats who received IT or IV PL had no significant differences in hemodynamic or morphometric measures, thus these data were combined. Hyperoxia-PL treated rats had significantly increased RV/LV+S, (Fig. 6A) and treatment with IV or IT WJ-MSC EVs attenuated RVH to a similar degree. Lung angiogenesis, pulmonary vascular remodeling, and lung alveolar structures were also similarly improved following IV or IT WJ-MSC EV delivery to hyperoxia-exposed rats (Fig. 6B–6F).

### WJ-MSC EVs Induce Pro-angiogenic Signaling Pathways

To further interrogate the mechanisms underlying the beneficial effects of WJ-MSC EVs in our experimental BPD and PH model, we evaluated the expression of pro-angiogenic genes in HULECs cultured in normoxic and hyperoxic conditions and exposed to WJ-MSC EVs or EV-depleted media. Compared to HULECs cultured in EV-depleted media, WJ-MSC EV treated hyperoxia-exposed HULECs had significant increases in eNOS (2.4-fold; *P* < .05), CXCL12 (2.2-fold; *P* < .05) and CXCR4 mRNA expression (2.4-fold; *P* < .05). While there were trends, there was no significant difference in Ang-1, HGF or VEGF expression in WJ-MSC EV treated HULECs.

We also evaluated the lung transcriptome of P14 rats who received medium-dose WJ-MSC EVs. Hyperoxia-PL rats had large transcriptomic differences relative to RA-PL rats on principal components (Supplementary Fig. S1A) and differential gene expression analyses (Supplementary Fig. S1B). Using a fold-change threshold of 2-fold in addition to the *P*- and *q*-values resulted in 2741 differentially expressed transcripts between Hyperoxia-PL compared to RA-PL and 2669 differentially expressed transcripts in WJ-MSC EV-treated hyperoxia exposed rats compared to RA-PL (Supplementary Fig. S1B). Comparison of EV-treated and PL-treated hyperoxia exposed rats revealed 77 differentially expressed transcripts, of which 46 were induced and 31 were suppressed (Supplementary Fig. S1C). Gene-set enrichment analysis of transcripts differentially expressed in EV-treated hyperoxia-exposed animals showed that induced transcripts were largely associated with pro-angiogenic pathways including a term for “blood vessel remodeling,” “blood vessel morphogenesis,” and “tube development.” In addition, induced genes were also associated with “regulation of intracellular calcium” a function critical for blood vessel smooth muscle contraction and “regulation of Notch signaling pathway” which regulates sprouting and branching morphogenesis (Supplementary Fig. S1D and S1E).

### Long-term Effects of a Single Dose of WJ-MSC EVs in BPD and PH

**Figure 7.**
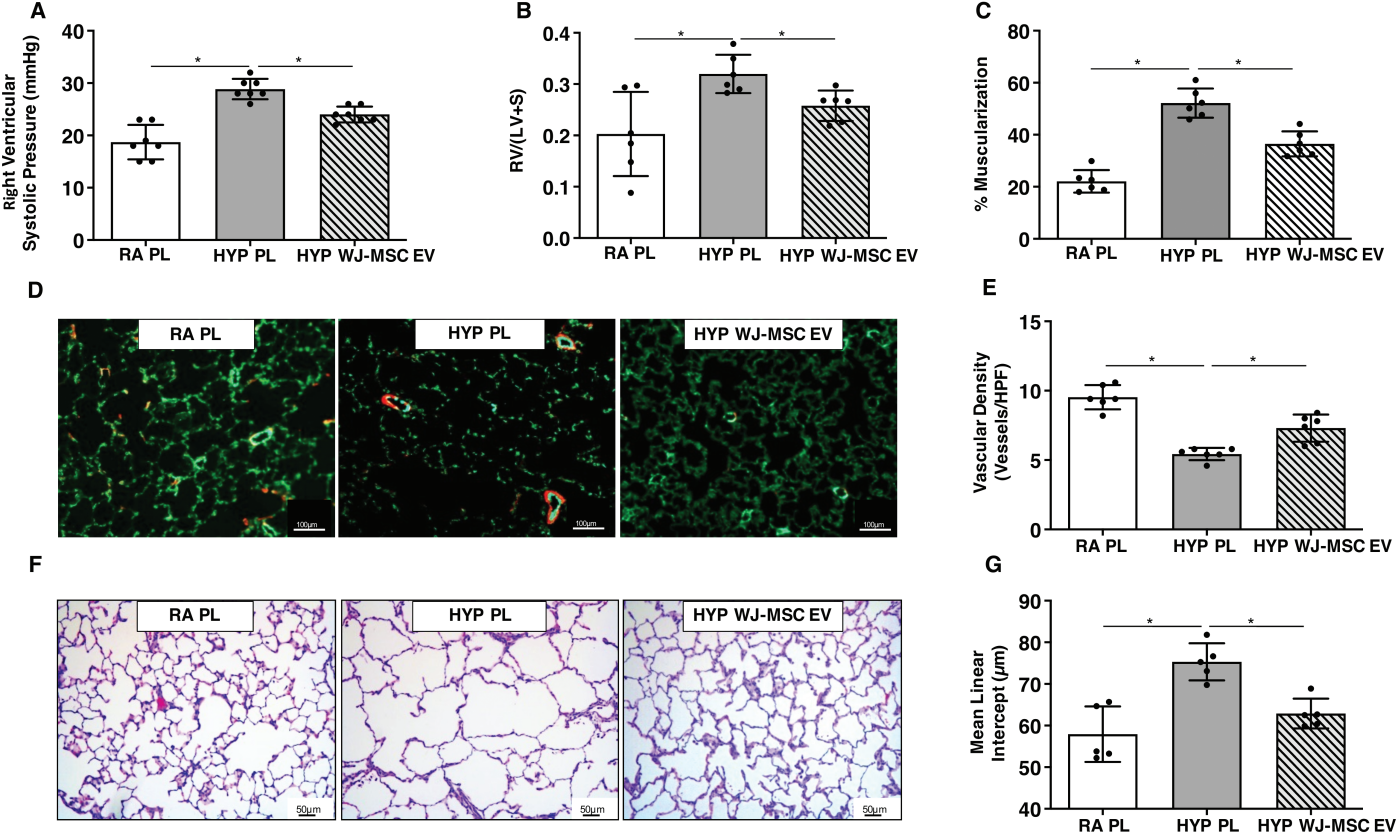
Long term effects of early IT WJ-MSC EVs in BPD and PH. Reduced (**A**) right ventricular systolic pressure, (**B**) RV/LV+S, and (**C**) % muscularized vessels in 3 month old hyperoxia (HYP) exposed rats treated with IT WJ-MSC EV at P3. (**D**) Lung sections stained with Von Willebrand Factor (green) and α-smooth muscle actin (red) demonstrating improved angiogenesis and decreased vascular remodeling in hyperoxia exposed rats treated with IT WJ-MSC EV. Original magnification 10×. (**E**) Increased lung vascular density in IT WJ-MSC EV hyperoxia-exposed rats. (**F**) H&E-stained lung sections demonstrating improved alveolar structure in 3 month old hyperoxia-exposed rats who received IT WJ-MSC EV. Original magnification ×. (**G**) Decreased mean linear intercept in hyperoxia-exposed IT WJ-MSC EV rats. Data are presented as mean ± SEM; *N* = 5–7/group. *P < .05, room air (RA) vs. HYP placebo (PL) or HYP placebo (PL) vs. HYP WJ-MSC EV.

Finally, since BPD and PH in preterm infants are associated with the persistent pulmonary vascular disease into adulthood,^35^ we questioned whether a single dose of WJ-MSC EVs in the neonatal period would have long-term lung protective effects. As there were no differences in the effects of WJ-MSC EVs at low, medium, or high doses, we used the medium dose of WJ-MSC EVs to evaluate the long-term effects of WJ-MSC EVs on lung structure and PH. Newborn rats exposed to hyperoxia from P1–P14 were given a single IT injection of WJ-MSC EVs on P3. Rats were recovered in room air from P14 to 3 months of age. Neonatal hyperoxia-induced persistent PH at 3 months of life, as evidenced by elevated RVSP in hyperoxia-PL treated animals (Fig. 7A). In contrast, treatment with a single dose of WJ-MSC EVs significantly attenuated PH, decreased RV hypertrophy, and reduced vascular remodeling, (Fig. 7A–7C). IT WJ-MSC EV treated rats also had greater preservation of lung vascular development and alveolar structure at 3 months of life as compared to hyperoxia-PL treated rats, (Fig. 7D–7G).

## Discussion

BPD complicated by PH continues to be a significant cause of morbidity and mortality.^36^ The balance between managing respiratory failure in the immediate post-natal period and protecting the preterm neonate from life-long cardiac and respiratory morbidity has been challenging. MSC EVs with their lung regenerative properties in experimental BPD have shown a promising new path toward achieving this goal. As with any new treatment modality, safety, optimal dosing, and long-term effects are equally important considerations along with efficacy to be scrutinized before taking the step toward clinical trials. In the present study, we attempted to answer translationally relevant questions. Ours is one of the first studies to use large-scale manufactured and GMP compliant clinical grade EVs^26^ and to the best of our knowledge, the first study to test varying doses and routes of administration of WJ-MSC EVs in experimental BPD. This study is also unique in presenting data on long-term cardio-pulmonary outcomes after IT WJ-MSC EV administration in the neonatal period.

First, our study establishes that IT administration is an effective route for the pulmonary delivery of MSC EVs. IT delivered MSCs have been previously shown to attenuate lung injury in adult lung injury^37^ and BPD-PH models.^12,13,26,38^ Interestingly, our ex-vivo tracking studies also detected the presence of IT-delivered MSC EVs in the liver and kidneys. This was surprising because although other studies have shown that IT delivered nanoparticles, can traffic from the lungs to lymph nodes and the bloodstream, these particles were smaller than our EVs.^39,40^ One possibility however is that the IT EVs were transported from the lungs within macrophages to other organs to exert systemic effects.^29,41^ This could be potentially advantageous as BPD and PH often co-exist with systemic organ dysfunction.

Another important question, which our study addressed, was the influence of MSC sources on EV efficacy in BPD-PH. Prior studies showed that WJ-MSCs have higher the proliferative capacity, greater differentiation potential, and higher expression of anti-inflammatory and angiogenesis-related growth factors as compared to BM-MSCs.^42-45^ In our study, while rats that received WJ-MSC EVs had less RVH and a trend for less mortality compared to those who received BM-MSC EVs, there was no significant difference in the lung alveolar and vascular structure. We acknowledge that the discrepancy in our findings and those of other investigators maybe secondary to our use of EVs from single donors and differences in our manufacturing protocols. Nonetheless, our present finding is similar to those of Willis et al who showed similar lung alveolar protective effects using BM or WJ-MSC EVs in a murine BPD model.^29^

We also investigated the optimal dosing of IT delivered WJ-MSC EVs for BPD-PH. Our findings suggest that the beneficial effects of WJ-MSC EVs on PH, vascular protection, and inflammation are non-dose dependents. This finding is important as it suggests that even at a low dosing, the EV cargo has pronounced lung protective effects. Our results are also consistent with other studies showing no difference in the lung regenerative effects of low, medium, or high dose MSCs.^11^ We however acknowledge that further work needs to be performed to elucidate whether cell equivalents, protein, or nanoparticle concentration is the ideal approach for MSC EV dosing and whether our current findings persist in long-term studies.^23^

Another important finding in our study was the long-term cardiopulmonary protective effects of a single dose of IT WJ-MSC EVs. Preterm infants, especially those with BPD and PH have persistent derangements in cardiopulmonary function extending long into adulthood. Our current finding extends those of Willis et al^46^ who demonstrated that intravenously delivered WJ-MSC EVs attenuate pulmonary vascular remodeling and reduce RVH in 28-day-old mice. Our model was however more severe, with follow-up at 3 months, which is equivalent to young adulthood. Moving forward it is critical to track the long-term fate of the EVs. One possibility is that the long-term beneficial effects are a consequence of the EVs altering endogenous stem cells or through epigenetic reprogramming of immune populations.^27,47^

One of the key questions that our present study also addressed is the comparative efficacy of systemically and locally administered MSC EVs for BPD-PH. Our study shows that systemically delivered WJ-MSC EVs have similar short-term beneficial effects on PH and lung angiogenesis as locally delivered MSC EVs. With the advent of less invasive ventilator strategies, our finding is important as systemically delivered EVs could also have beneficial effects on other organs injured by neonatal hyperoxia exposure. On the other hand, since preterm infants at the highest risk for BPD and PH are intubated, IT MSC EVs could be administered in much the same way as IT-instilled surfactants. Indeed, in a recently completed long-term follow-up of preterm patients who received IT MSCs for BPD prevention, IT MSC administration was shown to be safe.^48^ We however recognize that our present preclinical comparison is limited by short-term evaluations. Moving forward, long-term studies comparing systemic and locally delivered MSC-EVs along with multi-organ evaluations will be needed.

Finally, we also investigated the mechanisms by which WJ-MSC EVs exert their lung protective effects. Transcriptomic analysis revealed that WJ-MSC EVs modulate pathways involved in blood vessel remodeling, calcium transport, and blood vessel morphogenesis. The critical role of disordered vascular development in BPD and PH is well-recognized^49^ and prior studies have shown that MSC lung protective effects in BPD are in part mediated by pro-angiogenic factors, such as vascular endothelial growth factor (VEGF)^50^ and stromal-derived factor-1.^51^ In our current study, we also show that one of the key genes differentially expressed in hyperoxia-exposed WJ-MSC EVs treated rats was nephronectin, an extracellular matrix protein that is involved in tissue development and repair. While its role in BPD and PH is unknown, nephronectin modulates angiogenesis^52^ and genome-wide association studies suggest a potential association with COPD.^53^

## Conclusion

To the best of our knowledge, our study is one of the first to provide comprehensive insight into the ideal source, dosing, route, and long-term effects of GMP-grade MSC EVs for BPD and PH. We show that early administration of BM-MSC and WJ-MSC EVs have comparable lung protective and anti-inflammatory effects in experimental BPD and PH but WJ-MSC EVs prevent RV remodeling to a greater degree. We also show that a single WJ-MSC EV dose as low as 2 × 10^8^ particles per gram of body weight has significant beneficial effects in preventing the deleterious effects of hyperoxia on the developing lung. Moreover, systemically and locally delivered MSC-EVs attenuate PH to a similar degree. Importantly, the alveolar and pulmonary vascular regenerative effects of locally delivered WJ-MSC EVs persist into young adulthood. Together, our findings have significant translational implications for MSC EV therapies in preterm infants at high risk for BPD and PH as they provide a fundamental foundation for future clinical trials.

## Supplementary Material

szac041_suppl_Supplementary_File

## Data Availability

The data that support the findings of this study are available from the corresponding author upon reasonable request.
