# Supplementary material for: Mesenchymal Stem Cell-derived Extracellular Vesicles Prevent Experimental Bronchopulmonary Dysplasia Complicated By Pulmonary Hypertension
Source: Stem Cells Transl Med. 2022 Jun 27;11(8):828–40. doi: 10.1093/stcltm/szac041 (PMC9397655; doi:10.1093/stcltm/szac041)
Supplement: szac041_suppl_Supplementary_File [file szac041_suppl_supplementary_file.pdf]

SUPPLEMENTAL FILE

MESENCHYMAL STEM CELL DERIVED EXTRACELLULAR VESICLES PREVENT EXPERIMENTAL BRONCHOPULMONARY DYSPLASIA COMPLICATED BY PULMONARY HYPERTENSION

Mayank Sharma[1,2], Michael A. Bellio[3], Merline Benny[1,2], Shathiyah Kulandavelu[1,3], Pingping Chen[1,2], Janjindamai Chawisa[1,2], Shanique Sterling[1,2], Kevin Williams[1,2], Andreas Damianos[1,2], Sunil Batlahally[1,2], Kaitlyn Kelly[1,2], Daniela Aguilar-Caballero[1,2], Ronald Zambrano[1,2], Shaoyi Chen[1,2], Jian Huang[1,2], Shu Wu[1,2], Joshua M Hare[3,4], Augusto Schmidt[1,2], Aisha Khan[3], Karen Young[1,2, 3]

Supplemental Figure 1

A

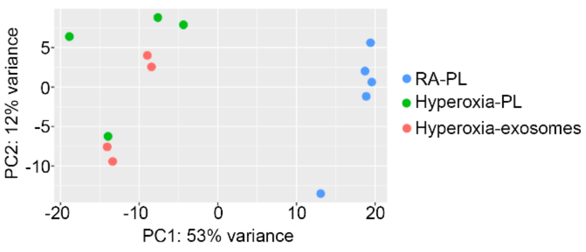

B

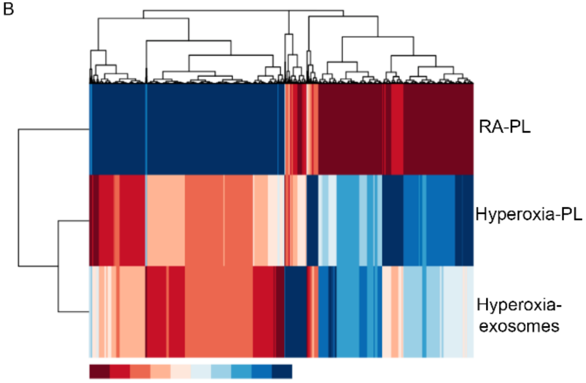

C

Gene name	log FC	FDR
Npnt	9.85	0.0000
Gsto1	8.35	0.0773
Mrtfa	7.86	0.0505
Ank2	6.92	0.0268
Slc10a6	4.64	0.0226
Epn2	1.98	0.0268
Plvap	1.12	0.0773
Pdzd2	1.11	0.0419
Pcdh17	1.10	0.0358
Sox7	1.10	0.0457
Ace	1.06	0.0113
Mettl24	1.04	0.0419
Pcdh1	1.02	0.0967
Adamtsl2	1.01	0.0996
Gpr182	0.98	0.0682
Krt15	-1.26	0.0996
Gramd1c	-1.30	0.0902
Lnpep	-1.45	0.0629
Mprip	-1.46	0.0268
Rhox5	-1.65	0.0106
Serpinb10	-1.68	0.0128
Nap1l5	-1.97	0.0090
Cyp2a1	-2.18	0.0037
Np4	-3.17	0.0840
RatNP-3b	-4.18	0.0185
Sema6c	-6.68	0.0652
Crem	-7.31	0.0902
Metrnl	-7.36	0.0013
Dnal1	-19.98	0.0004
Cldn7	-21.40	0.0000

D

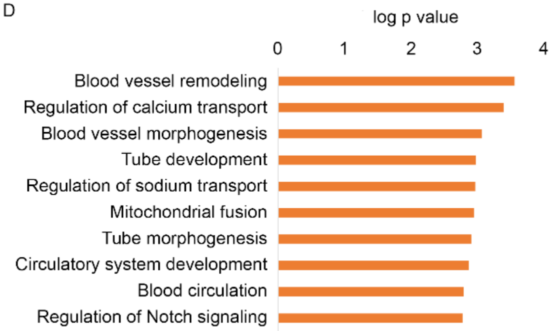

E

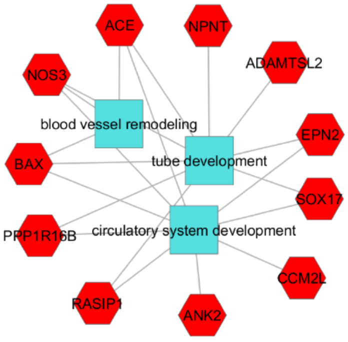

Supplemental Figure Legend

Supplemental Fig. 1

WJ-MSC EVs modulate lung angiogenic pathways

RNA-seq analysis of lungs from rats at P14 after exposure to room air (RA), hyperoxia placebo (PL), or hyperoxia WJ-MSC EVs show induction of pro-angiogenic pathways and processes in EV-treated rats relative to hyperoxia PL. (A) Principal component analysis shows clear separation of RA PL from hyperoxia exposed rats with partial overlap between hyperoxia PL and hyperoxia WJ-MSC EV rats. (B) Heatmap of differentially expressed transcripts show clustering of samples by treatment group. Hyperoxia PL animals showed 2741 differentially expressed transcripts compared to RA PL. Comparison of hyperoxia PL and hyperoxia WJ-MSC EV animals revealed 77 differentially expressed transcripts. (C) Top differentially expressed transcripts between hyperoxia WJ-MSC EVs and hyperoxia PL rats. (D) Gene set enrichment analysis of differentially expressed genes between hyperoxia PL and hyperoxia WJ-MSC EV rats shows induction of angiogenic processes and calcium metabolism. (E) Gene networks of induced genes associated with vascular development and remodeling.
